# Retraction: Socioeconomic shocks, social protection and household food security amidst COVID-19 pandemic in Africa’s largest economy

**DOI:** 10.1371/journal.pone.0335088

**Published:** 2025-10-23

**Authors:** The PLOS One Editors

The *PLOS One* Editors retract this article [1] because it was identified as one of a series of submissions for which we have concerns about potential manipulation of the publication process, peer review integrity, and authorship. These concerns call into question the validity and provenance of the reported results. We regret that the issues were not identified prior to the article’s publication.

RAO, OU, and AEI did not agree with the retraction. AHJ, JI, and AAO either did not respond directly or could not be reached.

## References

[1] Osabohien RA, Jaaffar AH, Ibrahim J, Usman O, Igharo AE, Oyekanmi AA. RETRACTED: Socioeconomic shocks, social protection and household food security amidst COVID-19 pandemic in Africa’s largest economy. PLoS One. 2024;19(1):e0293563. doi: 10.1371/journal.pone.0293563 38252674 PMC10802939

